# Anticancer and Immunomodulatory Effects of a Thiazolyl Benzodiazepine Targeting HSP90 in ER^+^ Breast Cancer

**DOI:** 10.3390/ph18111665

**Published:** 2025-11-04

**Authors:** Kubra Acikalin Coskun, Lutfi Tutar, Kezban Uçar Çifci, Mervenur Al, Irfan Koca, Mehmet Gumus, Levent Gulum, Emir Capkinoglu, Yusuf Tutar

**Affiliations:** 1 Division of Medicinal Biology, Department of Basic Medical Sciences, Istanbul Aydın University, Istanbul 34295, Turkey; kubraacikalincoskun@aydin.edu.tr; 2 Department of Molecular Biology and Genetics, Faculty of Science, Ahievran University, Kirsehir 40100, Turkey; lutfi.tutar@ahievran.edu.tr; 3 Hemp Research Institute, Yozgat Bozok University, Yozgat 66900, Turkey; kezban.u.cifci@bozok.edu.tr; 4 Division of Medicinal Biochemistry, Department of Basic Medical Sciences, University of Health Sciences, Istanbul 34668, Turkey; biochemistry.merve@gmail.com; 5 Division of Organic Chemistry, Department of Chemistry, Faculty of Science, Yozgat Bozok University, Yozgat 66900, Turkey; irfan.koca@bozok.edu.tr; 6 Program of Civil Defense and Firefighting, Yozgat Vocational School, Yozgat 66900, Turkey; mehmet.gumus@yobu.edu.tr; 7 Mudurnu Sureyya Astarcı Vocational School, Abant Izzet Baysal University, Bolu 14030, Turkey; leventgulum@ibu.edu.tr; 8 Department of General Surgery, Institute of Health Sciences, Acibadem Mehmet Ali Aydinlar University, Istanbul 34638, Turkey; 9 Division of Medicinal Biochemistry, Department of Basic Medical Sciences, Faculty of Medicine, Recep Tayyip Erdogan University, Rize 53100, Turkey; yusuf.tutar@erdogan.edu.tr; 10 Division of Biochemistry, Department of Basic Pharmaceutical Sciences, Faculty of Pharmacy, University of Health Sciences, Istanbul 34668, Turkey; 11 Molecular Oncology Division, Health Sciences Institutes, University of Health Sciences, Istanbul 34668, Turkey; 12 Personalized and Immunotherapy Practice and Research Center, University of Health Sciences, Istanbul 34668, Turkey; 13 Validebağ Experimental Medicine Practice and Research Center, University of Health Sciences, Istanbul 34668, Turkey

**Keywords:** HSP90 inhibition, thiazolyl benzodiazepine, cancer signaling, ERK/MAPK, pathway crosstalk

## Abstract

**Background:** Heat shock protein 90 (HSP90) is a molecular chaperone that stabilizes numerous oncogenic proteins and supports tumor survival. Small molecules targeting HSP90 offer a novel approach to overcome drug resistance and immune suppression in breast cancer. **Methods:** A novel thiazolyl benzodiazepine (TB) containing a hydrazone moiety was evaluated in breast cancer cell lines (ER^+^ MCF-7, TNBC MDA-MB-231, and HER2^+^ SK-BR-3). Cytotoxicity was assessed using the CCK-8 assay, followed by PCR sequencing, flow cytometry, RT-qPCR, protein profiling, and HSP90 binding assays. **Results:** TB showed the strongest activity in MCF-7 cells (IC_50_ = 7.21 µM) compared to MDA-MB-231 (IC_50_ = 28.07 µM) and SK-BR-3 (IC_50_ = 12.8 µM) cells. Mechanistic studies showed that TB binds to HSP90 (Kd = 3.10 µM), leading to disruption of the oncogenic signal. TB induced G2/M cell cycle arrest, promoted apoptosis via Bax and Caspase-3 activation, and suppressed cancer stem cell markers (*NANOG*, *OCT4*, *SOX2*). Additionally, TB activated immune-related pathways via ERK/MAPK signaling and upregulated genes such as *SMAD2, SMAD3,* and *JUN.* **Conclusions:** TB functions as an HSP90 inhibitor with dual anticancer and immunomodulatory properties in Estrogen Receptor-Positive (ER^+^) breast cancer cells. These findings suggest that TB represents a promising scaffold for the development of multi-targeted breast cancer therapies.

## 1. Introduction

Cancer remains one of the leading causes of death worldwide, and breast cancer contributes substantially to cancer incidence and mortality [1,2]. W. Coley, an American surgeon, discovered that administering vaccines from heat-killed bacteria to sarcoma patients might cause tumor regression, offering initial proof that inducing the immune system could be used for cancer treatment [3,4,5,6].

The development of novel medication therapies for safe and effective treatment of cancer is a never-ending battle. Chemotherapy continues to be the mainstay of cancer treatment, in addition to the use of surgery and radiation. However, the need for alternative strategies is evident, particularly approaches that can modulate the immune system while directly targeting cancer cell survival pathways. The immune system employs a diverse range of cells and pathways to reduce or promote cellular immunity and this is the basis of small molecule immune treatments for cancer. Novel cancer treatment approaches are critical for immunotherapy to advance [7].

As a result, interest in developing small molecule-based immunotherapies, which offer distinct advantages such as oral bioavailability, improved tumor penetration, and access to intracellular targets, is growing [7]. Despite their potential, small molecule immunotherapies are still underdeveloped, and there is a clear lack of knowledge regarding compounds with dual anticancer and immunomodulatory effects.

Significant efforts have been made in the design and discovery of new anticancer drugs that center on the benzodiazepine template [8,9,10].

Among potential targets, heat shock protein 90 (HSP90) functions as a molecular chaperone that stabilizes numerous oncogenic client proteins and supports immune suppression, making it an attractive therapeutic candidate. Inhibition of HSP90 offers the possibility of simultaneously disrupting multiple signaling pathways, thereby overcoming resistance and enhancing antitumor immune responses [1,7,11].

**Figure 1:**
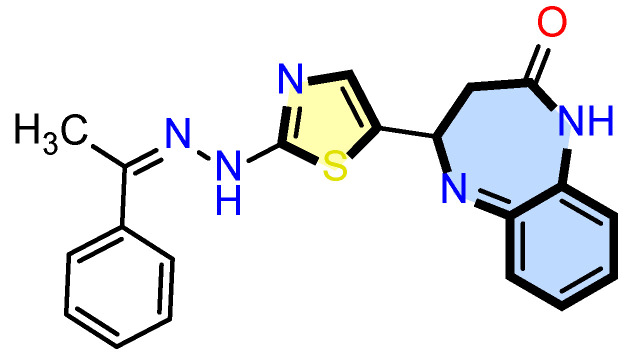
Thiazolyl benzodiazepine (TB).

One strategy increasingly adopted in oncology is the development of multifunctional molecules that combine distinct pharmacophores within a single scaffold. This approach allows simultaneous modulation of altered molecular networks, potentially improving efficacy while reducing toxicity. In line with this rationale, our group designed a novel compound by fusing a benzene and diazepine ring with a thiazole and hydrazone moiety, generating a thiazolyl benzodiazepine (TB) (Figure 1) [11].

Benzodiazepine derivatives have recently emerged as promising scaffolds with anticancer potential [7]. Since benzodiazepine derivatives have shown promising activity in distinct cancer cell types, we tested thiazolyl benzodiazepine (TB) in ER^+^ MCF-7 breast cancer cells. This work focuses on a TB which displays promising anticancer activity and molecular mechanism of the compound screened. The TB, as an HSP90 inhibitor, displays a promising template for anticancer drug development.

## 2. Results

### 2.1. Cell Cytotoxicity Assay

**Figure 2:**
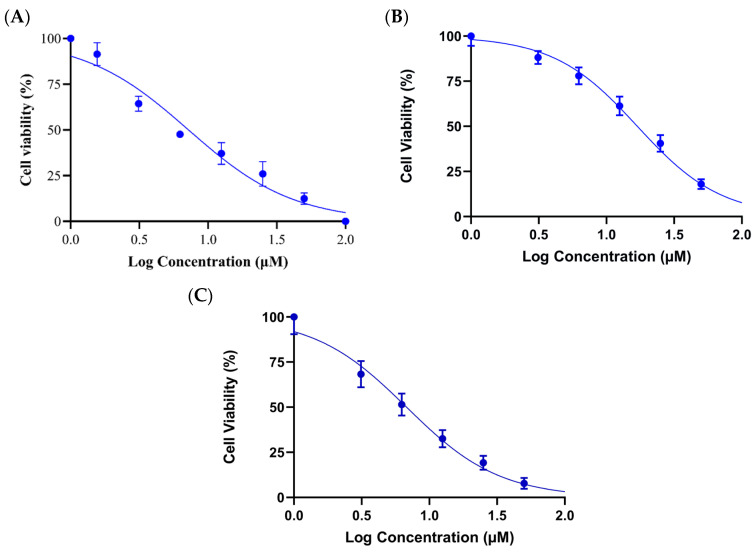
Cytotoxic effect of TB on breast cancer cell lines. (**A**) MCF-7 (ER^+^), (**B**) MDA-MB-231 (triple-negative), (**C**) SK-BR-3 (HER2^+^) cells were treated with TB.

The TB compound was evaluated against Estrogen Receptor-Positive Human Breast Cancer (MCF-7), Triple-Negative Human Breast Cancer (MDA-MB-231), and HER2-Positive Human Breast Cancer (SK-BR-3) cell lines by CCK-8 assays and was found to have significant anticancer activity in the MCF-7 cell line. TB causes cytotoxicity in MCF-7, MDA-MB-231, and SK-BR-3 cells with half maximal inhibitory concentration (IC_50_) values of 7.21 μM, 28.07 μM, and 12.8 μM, respectively (Figure 2). The compound was dissolved in DMSO (0.1%) and the same amount was added to control cells to eliminate any potential artifact signal.

Since the cytotoxicity of TB was highly active in the MCF-7 cell line (7.2 µM), it was chosen as a model for further experiments.

### 2.2. Array Studies and Gene Enrichment Analysis

**Figure 3:**
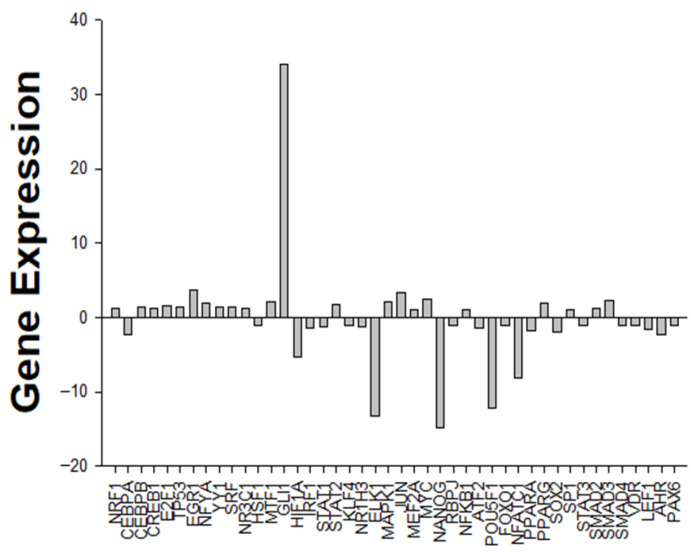
Expression enhancement of array genes in the presence of TB in MCF-7 cell lines.

To explore the transcriptional changes induced by TB, a focused RT^2^ Profiler PCR array was used to evaluate the expression of genes related to apoptosis, inflammation, and immune signaling in MCF-7 cells. The bar graph in Figure 3 displays the fold change in expression levels of individual genes compared to untreated controls, revealing distinct upregulation and downregulation patterns.

Among the most strongly upregulated genes were JUN, *SMAD3*, and *MAPK1*, while several pro-survival or stemness-associated genes, including *NANOG* and *OCT4*, were markedly downregulated. These data indicate that TB modulates a wide array of molecular targets associated with both pro-apoptotic signaling and immune system activation.

**Figure 4:**
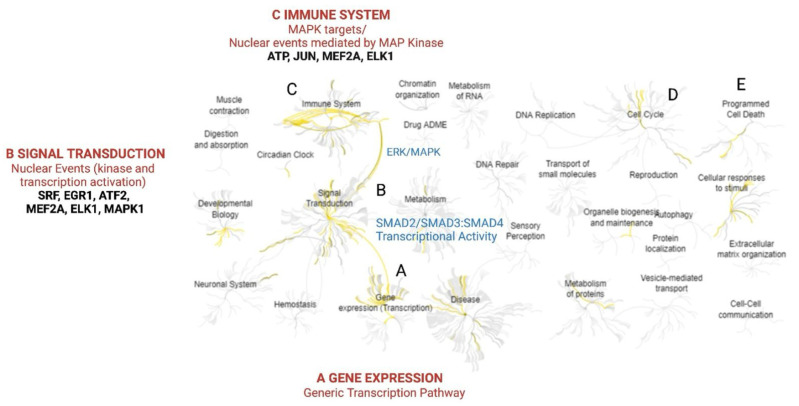
Gene enrichment analysis of TB-treated MCF-7 cell lines. Mechanism of action of TB is shown by A–C. TB induces a set of transcription factors and transduces signals via *SMAD* (**A**). Through ERK/MAPK signaling, the immune system is triggered (**B**). ATP, *JUN*, *MEF2A*, and *ELK1* play important roles in immune system induction (**C**). Inhibiting cancer cell-associated pathways potentially results in intrinsic apoptosis as shown by gene enrichment analysis. Gene enrichment analysis was performed using Reactome pathway database, with significance cutoff at *p* < 0.05; the visualization was modified by BioRender. Tutar, Y. (2025) https://BioRender.com/q48f439.

To further interpret the biological significance of these transcriptional changes, gene enrichment analysis was conducted using pathway databases. As visualized in Figure 4, TB appears to engage interconnected pathways including the ERK/MAPK, SMAD-dependent transcription, and immune-related cascades. These interconnected events are likely contributors to the observed phenotypes such as apoptosis and cell cycle arrest (explored further in Section 2.3, Section 2.4 and Section 2.5).

### 2.3. Cell Cycle Analysis

**Figure 5:**
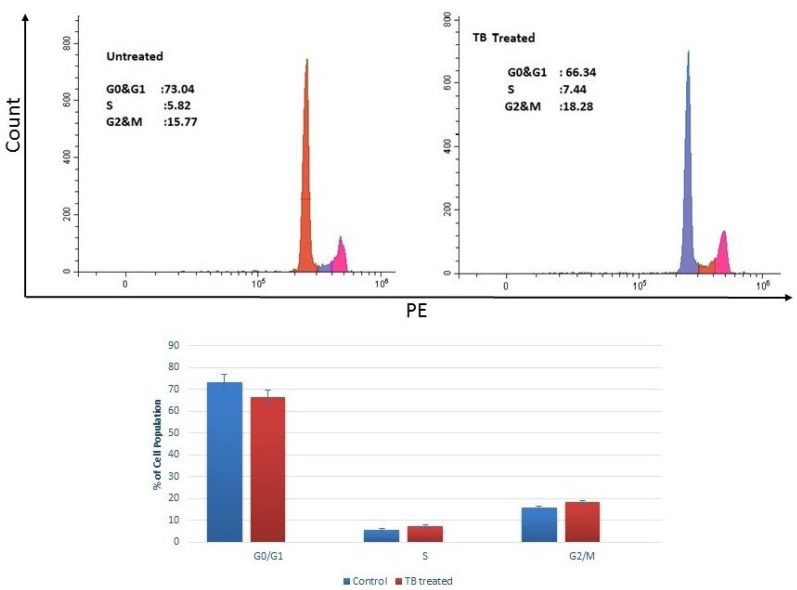
Cell cycle analysis of TB-treated cells. TB induces G2/M phase arrest in ER^+^ breast cancer cells.

Flow cytometry analysis demonstrated that TB treatment altered the distribution of cell cycle phases in MCF-7 cells, leading to a modest accumulation in the G2/M phase. Specifically, the proportion of cells in the G2/M phase increased from 15.77% in control cells to 18.28% in TB-treated cells, while the G1 population decreased from 73.04% to 66.34% (Figure 5). These results suggest that TB induces G2/M phase arrest, impairing normal cell cycle progression.

To further understand the molecular mechanisms behind this arrest, gene and protein expression analyses were performed (Section 2.4 and Section 2.5).

### 2.4. Gene Expression Profiling

**Figure 6:**
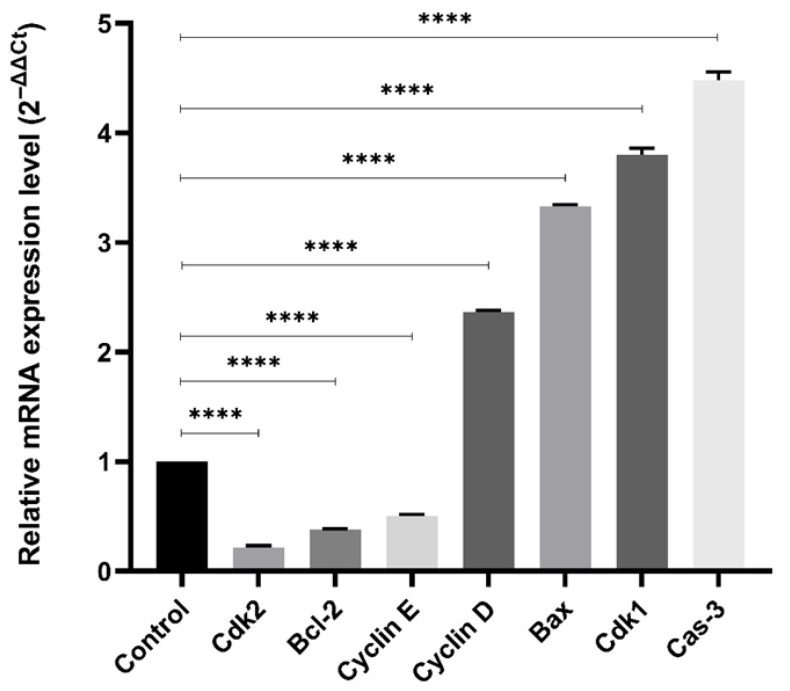
Gene expression analysis of apoptosis- and cell cycle-related genes after TB treatment. Expression levels were normalized to control. (n = 3; **** *p* < 0.0001).

To support the flow cytometry findings and explore the molecular drivers of cell cycle arrest and apoptosis, we analyzed the expression of cell cycle and apoptotic genes by RT-qPCR (Figure 6).

TB treatment increased the expression of Cyclin D and CDK1, which are key regulators of the G2/M transition, while Cyclin E and CDK2, associated with G1/S progression, were downregulated. These transcriptional changes are consistent with the G2/M arrest observed in flow cytometry (Section 2.3).

In addition, apoptosis-related genes were also evaluated. Pro-apoptotic Bax and Caspase 3 were upregulated, whereas anti-apoptotic Bcl2 was significantly downregulated, indicating activation of the mitochondrial apoptosis pathway. Protein level confirmation of these findings is provided in Section 2.5.

### 2.5. Protein Expression Profiling

**Figure 7:**
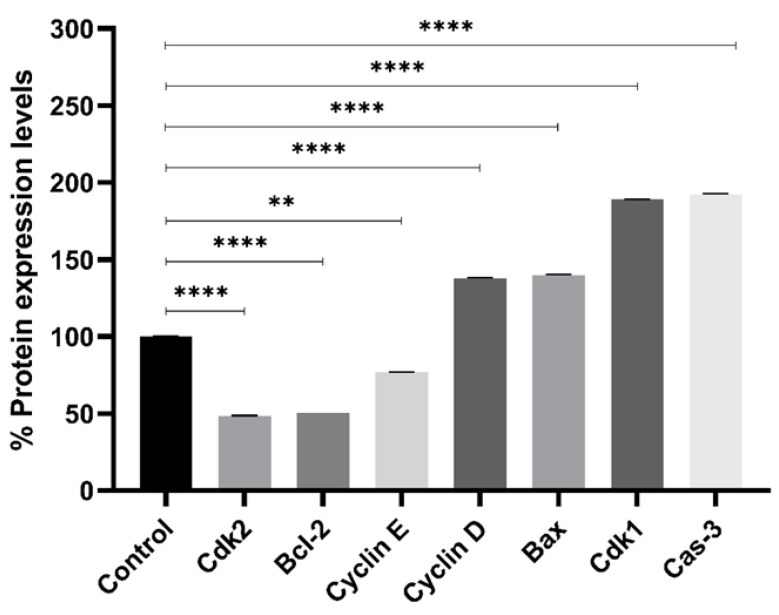
Protein expression profiles of cell cycle and apoptosis intermediates in the presence and absence of TB (n = 3, ** *p* < 0.01, **** *p* < 0.0001).

To validate the transcriptional findings at the translational level, Western blot analysis was performed for selected cell cycle and apoptotic proteins in TB-treated MCF-7 cells (Figure 7).

Consistent with the RT-qPCR results (Section 2.4), TB treatment resulted in upregulation of Cyclin D1 and CDK1, which are associated with G2/M checkpoint progression, and downregulation of Cyclin E and CDK2, involved in G1/S transition. These findings further corroborate the G2/M arrest observed in flow cytometry (Section 2.3).

In parallel, the expression of apoptosis-related proteins was also evaluated. Pro-apoptotic markers Bax and cleaved Caspase-3 were significantly increased, while the anti-apoptotic Bcl-2 protein was decreased in response to TB. This confirms the activation of the intrinsic (mitochondrial) apoptotic pathway at the protein level and aligns with the gene expression and flow cytometry data.

Altogether, the coordinated changes in gene and protein expression, along with functional flow cytometry data, support the conclusion that TB induces apoptosis and cell cycle arrest through the modulation of key regulatory pathways.

### 2.6. Binding Assay

**Figure 8:**
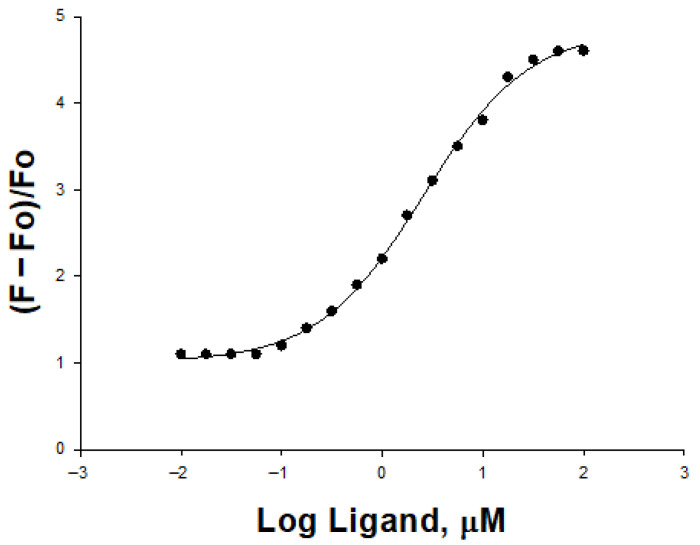
Binding of TB to HSP90 protein using ANS displacement assay. 8-Anilinonaphthalene-1-sulfonic acid (ANS) was used as a fluorescent probe to determine binding affinity.

To understand the effect of TB on inhibition, HSP90 binding was performed as the protein interacts with several signaling proteins at the cancer pathway. The binding constant was calculated as 3.10 µM (Figure 8). TB binding indicates that the compound perturbs the function of HSP90. HSP90 has several substrate proteins that help cancer cell survival with several mechanisms. Immune system suppression is one important function of cancer cells. TB binding to HSP90 perturbs its function and destabilizes HSP90 substrate oncoprotein. At the same time, this action activates the immune system (Figure 4). Off-target effects of TB have not been characterized but the Hsp90 client protein most likely helps immune system suppression and perturbing the HSP90 conformation by TB destabilizes the client protein and/or results in it not being able to fold its native structure to gain full function. This may boost the immune function over oncogene-suppressed pathways in cancer cells. Thus, HSP90-TB binding reduces cancer activity and induces the immune system.

## 3. Discussion

Although TB also induced cytotoxicity in triple-negative MDA-MB-231 (IC_50_ 28.07 µM) and HER2^+^ SK-BR-3 (IC_50_ 12.8 µM) cells, its significantly higher activity in ER^+^ MCF-7 (IC_50_ 7.2 µM) cells led us to focus our mechanistic investigations on this line. This selectivity suggests that the HSP90-mediated activity of TB may be potentiated by estrogen receptor-related signaling and luminal transcription programs.

Cell signaling regulates many physiological responses, and HSPs activate key oncogenic pathways. Our study indicates that inhibition of these proteins can block cancer cell proliferation and differentiation. This aligns with the rationale that targeting common factors within cancer pathways can augment drug efficacy, reduce dosage, and lower toxicity while delaying resistance.

Recent data from our group showed that small molecules can inhibit both HSP70 and HSP90 simultaneously at lower concentrations. In TB-treated MCF-7 cells, mRNA expression analysis revealed activation of the ERK/MAPK pathway. This pathway not only regulates inflammation and cell differentiation but also plays a role in apoptosis and cell cycle control. Although the IC_50_ values of TB are relatively high compared to classical HSP90 inhibitors, the compound’s ability to bind HSP90 (Kd = 3.10 µM) and regulate downstream target-related signaling pathways supports its target-specific mode of action. Given that TB is structurally distinct from traditional inhibitors, it may interact with alternative binding domains on HSP90, potentially leading to partial or indirect inhibition and allowing for simultaneous modulation of multiple intracellular pathways. Therefore, the TB scaffold holds promise for future structural optimization efforts aimed at generating more potent analogs.

Previous reports have shown that the degree of ERK/MAPK activation correlates with prognosis in breast cancer [12,13] and contributes to chemoresistance [14,15]. Our results confirm that TB interferes with this signaling cascade, leading to apoptosis and G2/M cell cycle arrest, as supported by both array and flow cytometry analyses.

Cancer cells often suppress immune responses to favor survival. Interestingly, TB treatment activated multiple immune-related pathways, including TRAF6-mediated NF-κB induction, IRF7 activation via TLR7/8 or TLR9, TRIF-mediated TLR4 signaling, and MyD88-dependent and -independent cascades. Enrichment analysis also highlighted the involvement of interleukin -4, -13, and -17 signaling, all of which play critical roles in tumor–immune interactions [16,17]. Together, these findings indicate that TB may simultaneously target oncogenic signaling and may modulate immune-related signaling pathways. The link between HSP90 inhibition and immune pathway activation is based solely on PCR array and enrichment analysis. Further, potential off-targets were not investigated in the study.

Classical HSP90 inhibitors such as geldanamycin and its derivative 17-AAG have shown strong anticancer effects but are limited by hepatotoxicity, poor solubility, and unfavorable pharmacokinetic profiles. By contrast, TB, as a thiazolyl benzodiazepine scaffold, may offer potential advantages as a small molecule. Its strong cytotoxicity in ER^+^ breast cancer cells, combined with immune pathway modulation, suggests a dual mechanism of action [18]. These advantages may be attributed to structural differences. Geldanamycin and its analogs (e.g., 17-AAG, 17-DMAG) contain benzoquinone moieties that undergo redox cycling, leading to reactive oxygen species (ROS) generation and hepatotoxicity [19]. Their bulky macrocyclic and hydrophobic structures also cause poor aqueous solubility and low bioavailability, complicating formulation and delivery [20]. In contrast, TB is a non-quinone, low-molecular-weight thiazolyl benzodiazepine with improved drug-like properties. These features may reduce redox-related toxicity and enhance pharmacokinetic behavior. Furthermore, its scaffold allows for synthetic flexibility, enabling the development of optimized analogs with improved safety and efficacy profiles. Furthermore, unlike antibody-based immunotherapies which suffer from high cost, immunogenicity, and limited tissue penetration, TB possesses drug-like properties that may allow oral administration, a key advantage of small-molecule immunomodulators. Taken together, TB may serve not as a replacement but as a complementary strategy to existing anticancer therapies, with potential utility in combination regimens to enhance efficacy and reduce toxicity.

Another important observation is TB’s ability to suppress cancer stem cell markers. Cancer stem cells are known for their self-renewal capacity and differentiation potential, which contribute to therapeutic resistance and tumor recurrence. By targeting these cells, TB may overcome one of the critical limitations of many standard therapies. This unique property supports its potential as a lead compound for anticancer drug development.

While our study provides mechanistic insights into the anticancer activity of TB, it has limitations. The analyses were largely restricted to in vitro experiments, with mechanistic studies focused on MCF-7 cells. Future research should include validation in in vivo animal models to assess therapeutic efficacy, pharmacokinetics, and systemic toxicity. In addition, extending functional assays to resistant phenotypes and diverse breast cancer subtypes will be important to confirm the broader translational relevance of TB.

Another limitation of the current study is the absence of non-malignant breast epithelial control cell lines (e.g., MCF-10A), which prevents a comprehensive assessment of TB’s selectivity and off-target toxicity. However, as this work aimed to explore the mechanistic anticancer and immunomodulatory effects of TB in ER^+^ breast cancer cells where the compound demonstrated the strongest activity, non-malignant lines were not included at this stage. Future studies will include non-malignant control cell lines to evaluate selectivity and better define the therapeutic index of TB.

## 4. Materials and Methods

### 4.1. Cell Culture, PCR Array Studies and Gene Enrichment Analysis

Breast cancer cells MCF-7 (ATCC^®^ HTB-22), MDA-MB-231 (ATCC^®^ HTB-26), and SK-BR-3 (ATCC^®^ HTB-30) were cultured in DMEM (Dulbecco’s Modified Eagle’s Medium; Sigma-Aldrich, Burlington, MA, USA) containing 10% heat-inactivated fetal bovine serum, 1% l-glutamine, 100 IU/mL penicillin, and 10 mg/mL streptomycin (Gibco, Thermo Fisher Scientific, Waltham, MA, USA) in 75 cm^2^ polystyrene flasks. Cells were cultured at 37 °C in a humidified atmosphere of 5% CO_2_ [2]. The effects of thiazolyl benzodiazepine (TB) on MCF-7, MDA-MB-231, and SK-BR-3 viability were assessed using the CCK-8 assay (Sigma-Aldrich, Burlington, MA, USA). MCF-7 (5 × 10^3^ per well) were seeded in 96-well plates. After incubation overnight, supernatants were replaced with conditional medium containing different concentrations of thiazolyl benzodiazepine (TB) (1.56 μM, 3.125 μM, 6.25 μM, 12.5 μM, 25 μM, 50 μM, 100 μM). Following with culture for 48 h, 10 μL CCK-8 solution was added (96992; Sigma-Aldrich) and incubated for 4 h at 37 °C. The absorbance was measured at 450 nm using a microplate reader (Thermo Fisher Scientific, Waltham, MA, USA).

MCF-7 cells were incubated with TB, and total mRNA were extracted and converted to cDNA. PCR array experiments were carried out on MCF-7 cells to elucidate breast cancer gene expression alteration in the presence of TB and all experiments were performed three times. TB was incubated with cancer cells for 48 h. The compound was dissolved in 0.1% DMSO (Sigma-Aldrich, Burlington, MA, USA) and same amount was used in control experiments to eliminate any interference. After the cells reached 80% confluence, TB was applied on the cells for 48 h. After incubation, total RNA (Analytica Jena, Jena, Germany) was isolated and first strand cDNA (Applied Biological Materials, Richmond, BC, Canada) was synthesized. Custom-designed PCR cancer arrays were used in this experiment. Primers were purchased from Sigma-Aldrich. RT-PCR experiments were performed in Analytik Jena qTOWER3 instrument by using SYBR Green Master Mix (Euroclone, Pero, Italy). Experiments were performed using 1 µg total RNA and SYBR Green Master Mix (Euroclone, Pero, Italy) for 95 °C 15 s, 60 °C 30 s, 95 °C 5 min, 40 cycles. The analysis was repeated in three replicates and the expression levels of the genes were determined by the 2^−ΔΔCt^ method [2,21,22].

In MDA-MB-231 and SK-BR-3 cells, only Cell Counting Kit-8 (CCK-8) viability assay was performed to compare the efficacy of TB in different molecular subtypes of breast cancer. PCR arrays, cell cycle analysis by flow cytometry, RT-qPCR, and protein level studies were performed in MCF-7 cells where TB was most effective (IC_50_ = 7.21 µM).

### 4.2. Cell Cycle Analysis

The analysis was carried out according to the MAK344 Cell Cycle Analysis kit. MCF-7 cells (3 × 10^5^ cells/well) were treated and prepared with the kit protocol. Then, the cells were analyzed using flow cytometry (Beckman Coulter, Cytoflex, Indianapolis, IN, USA) [21,22].

### 4.3. Gene Expression Profiling

To determine the gene expression levels of Cyclin D, Cyclin E, Bcl-2, Caspase-3, CDK1, CDK2, and Bax cells were treated with TB at IC value for 48 h, and at the end of the period, RNA isolation and cDNA synthesis were performed from the collected cells. The primers used are given in the Supplementary Data. The GADPH gene was used as a reference gene. Gene expressions were calculated by the 2^−ΔΔCt^ method [21,22].

### 4.4. Protein Expression Profiling

Cells were treated with TB for 48 h, and at the end of the period, the collected cells were tested using the Biuret method in Lysis Buffer. CCND1, CDK1, Bcl-2, Caspase-3, and Cas-9 protein levels were measured using the Mybiosource Human Elisa Kit (MyBioSource, San Diego, CA, USA) [23].

### 4.5. Binding Assay

HSp90 protein purification and binding assay were performed according to previously established protocols. Briefly, HSP90α plasmid (Applied Biological Materials Inc., Richmond, BC, Canada) was transformed into BL21 (DE3) competent cells and expressed four hours after IPTG induction. Then, the protein was purified with DEAE weak anion resin at phosphate buffer. Since the intrinsic signal of HSP90 is lower, external probe ANS was employed to calculate the Kd value [2,24].

### 4.6. Statistical Analysis

All experiments were performed in biological triplicates (n = 3) unless otherwise specified. Data are presented as mean ± standard deviation, and statistical significance was evaluated using Student’s *t*-test or one-way ANOVA using GraphPad Prism software (version 8.0; GraphPad Software, San Diego, CA, USA). Probability values *p* < 0.05 were considered statistically significant. IC_50_ values were calculated using nonlinear regression (log[inhibitor] vs. response–variable slope, four-parameter logistic model) in GraphPad Prism 8.0.

## 5. Conclusions

To conclude, understanding the cellular and molecular mechanisms of various kinds of cell death has advanced significantly in recent decades. These discoveries have revealed molecular abnormalities in these pathways in a variety of cancer types, suggesting possible candidates for more potent treatment. Since the goal of cancer therapy is to cause cancer cells to die, understanding how to regulate the cell cycle has created a number of possibilities for cutting-edge cancer treatments. Therefore, the compound may serve as a potential therapeutic lead in breast cancer but may provide therapeutic benefit either as a single approach or in combination with established clinical drugs, since the compound inhibits the p38 MAPK pathway and the pathway’s crosstalk with other pathways and receptors. Having more treatment targets could facilitate HSP inhibitory combination therapies in distinct cancer types and may further prevent chemoresistance and lower effective drug dosage.

## Data Availability

The data presented in this study are contained within the article and its Supplementary Materials. Further inquiries can be directed to the corresponding author. Genes employed in this study are given at Supplementary Data with Ensembl (ensemble.org) and Hugo Gene Nomenclature Committee-HGNC (genenames.org) identification numbers.

## Supplementary Materials

The following supporting information can be downloaded at: https://www.mdpi.com/article/10.3390/ph18111665/s1, Primer Sequences and Genes List with Ensembl and Hugo Gene Nomenclature Committee-HGNC identification numbers.

## Abbreviations

The following abbreviations are used in this manuscript:

HSP	Heat shock protein
TB	Thiazolyl benzodiazepine
MCF-7	Human breast epithelial carcinoma
ATCC	American Type Culture Collection
DMEM	Dulbecco′s Modified Eagle′s Medium
CCK-8	Cell Counting Kit-8
HSP90	Heat Shock Protein 90
ER^+^	Estrogen Receptor-Positive
IC_50_	Half Maximal Inhibitory Concentration
MAPK	Mitogen-Activated Protein Kinase
MDA-MB-231	Triple-Negative Human Breast Cancer Cell Line
SK-BR-3	HER2-Positive Human Breast Cancer Cell Line
