# Supplementary material for: Anticancer and Immunomodulatory Effects of a Thiazolyl Benzodiazepine Targeting HSP90 in ER+ Breast Cancer
Source: Pharmaceuticals (Basel). 2025 Nov 4;18(11):1665. doi: 10.3390/ph18111665 (PMC12655246; doi:10.3390/ph18111665)
Supplement: Supplementary file 1 [file pharmaceuticals-18-01665-s001.zip › pharmaceuticals-3891427-supplementary.pdf]

Supplementary Data

Primer Sequences

**Primer**	**Sequence (5'-3')**
Cyclin D-F	AGACCTGCGCGCCCTCGGTG
Cyclin D-R	GTAGTAGGACAGGAAGTTGTTC
Cyclin E -F	GTCCTGGCTGAATGTATACATGC
Cyclin E- R	CCCTATTTTGTTCAGACAACATGGC
CDK1-F	GCGGAATAATAAGCCGGGAT
CDK1-R	CAACTCCATAGGTACCTTCTCCA
CDK2-F	CCAGGAGTTACTTCTATGCCTGA
CDK2-R	TTCATCCAGGGGAGGTACAAC
Bax-F	TCAGGATGCGTCCACCAAGAAG
Bax-R	TGTGTCCACGGCGGCAATCATC
Cas3-F	GGAAGCGAATCAATGGACTCTGG
Cas3-R	GCATCGACATCTGTACCAGACC
Bcl-2-F	ATCGCCCTGTGGATGACTGAGT
Bcl-2-R	GCCAGGAGAAATCAAACAGAGGC
GADPH-F	GTCTCCTCTGACTTCAACAGCG
GADPH-R	ACCACCCTGTTGCTGTAGCCAA

Genes employed in the study with Ensembl (ensemble.org) and Hugo Gene Nomenclature Committee-HGNC (genenames.org) identification numbers:

ACTB, actin beta, ENSG00000075624, HGNC ID: 132

AHR, aryl hydrocarbon receptor, ENSG00000106546, HGNC ID: 348

ATF2, activating transcription factor 2, ENSG00000115966, HGNC ID:784

CEBPA, CCAAT enhancer binding protein alpha, ENSG00000245848, HGNC ID: 1833

CEBPB, CCAAT enhancer binding protein beta, ENSG00000172216, HGNC ID: 1834

CREB1, cAMP responsive element binding protein 1, ENSG00000118260, HGNC ID: 2345

E2F1, E2F transcription factor 1, ENSG00000101412, HGNC ID: 3113

EGR1, early growth response 1, ENSG00000120738, HGNC ID: 3238

ELK1, ETS transcription factor ELK1, ENSG00000126767, HGNC ID: 3321

FOXO1, forkhead box O1, ENSG00000150907, HGNC ID: 3819

GAPDH, glyceraldehyde-3-phosphate dehydrogenase, ENSG00000111640, HGNC ID: 4141

GATA1, GATA binding protein 1, ENSG00000102145, HGNC ID: 4170

GLI1, GLI family zinc finger 1, ENSG00000111087, HGNC ID: 4317

HIF1A, hypoxia inducible factor 1 subunit alpha, ENSG00000100644, HGNC ID: 4910

HNF4A, hepatocyte nuclear factor 4 alpha, ENSG00000101076, HGNC ID: 5024

HSF1, heat shock transcription factor 1, ENSG00000185122, HGNC ID: 5224

IRF1, interferon regulatory factor 1, ENSG00000125347, HGNC ID: 6116

JUN, Jun proto-oncogene, AP-1 transcription factor subunit, ENSG00000177606, HGNC ID: 6204

KLF4, KLF transcription factor 4 , ENSG00000136826, HGNC ID: 6348

LEF1, lymphoid enhancer binding factor 1, ENSG00000138795, HGNC ID: 6551

MAPK1, mitogen-activated protein kinase 1, ENSG00000100030, HGNC ID: 6871

MEF2A, myocyte enhancer factor 2A, ENSG00000068305,HGNC ID: 6993

MTF1, metal regulatory transcription factor 1, ENSG00000188786, HGNC ID: 7428

MYC, MYC proto-oncogene, bHLH transcription factor, ENSG00000136997, HGNC ID: 7553

NANOG, Nanog homeobox, ENSG00000111704, HGNC ID: 20857

NFATC1, nuclear factor of activated T cells 1, ENSG00000131196, HGNC ID: 7775

NFKB1, nuclear factor kappa B subunit 1, ENSG00000109320, HGNC ID: 7794

NFYA, nuclear transcription factor Y subunit alpha, ENSG00000001167, HGNC ID: 7804

NR1H3, nuclear receptor subfamily 1 group H member 3, ENSG00000025434, HGNC ID: 7966

NR3C1, nuclear receptor subfamily 3 group C member 1, ENSG00000113580, HGNC ID: 7978

NRF1, nuclear respiratory factor 1, ENSG00000106459, HGNC ID: 7996

PAX6, paired box 6, ENSG00000007372, HGNC ID: 8620

POU5F1, POU class 5 homeobox 1, ENSG00000204531, HGNC ID: 9221

PPARA, peroxisome proliferator activated receptor alpha, ENSG00000186951, HGNC ID: 9232

PPARG, peroxisome proliferator activated receptor gamma, ENSG00000132170, HGNC ID: 9236

RBPJ, recombination signal binding protein for immunoglobulin kappa J region, ENSG00000168214, HGNC ID: 5724

SMAD2, SMAD family member 2, ENSG00000175387, HGNC ID: 6768

SMAD3, SMAD family member 3, ENSG00000166949, HGNC ID: 6769

SMAD4, SMAD family member 4, ENSG00000141646, HGNC ID: 6770

SOX2, SRY-box transcription factor 2, ENSG00000181449, HGNC ID: 11195

SP1, Sp1 transcription factor, ENSG00000185591, HGNC ID: 11205

SR, serum response factor, ENSG00000112658, HGNC ID: 11291

STAT1, signal transducer and activator of transcription 1, ENSG00000115415, HGNC ID: 11362

STAT2, signal transducer and activator of transcription 2, ENSG00000170581, HGNC ID: 11363

STAT3, signal transducer and activator of transcription 3, ENSG00000168610, HGNC ID: 11364

TP53, tumor protein p53, ENSG00000141510, HGNC ID: 11998

VDR, vitamin D receptor, ENSG00000111424, HGNC ID: 12679

YY1, YY1 transcription factor, ENSG00000100811, HGNC ID: 12856
